# Osteosarcoma cell proliferation and migration are partly regulated by redox-activated NHE-1

**Published:** 2015-12-12

**Authors:** Hua Bai, Guojing Chen, Congwen Fang, Xuekang Yang, Sixun Yu, Chunxu Hai

**Affiliations:** 1 Department of Toxicology, Shaanxi Provincial Key Lab of Free Radical Biology and Medicine, the Ministry of Education Key Lab of Hazard Assessment and Control in Special Operational Environment, School of Public Health, Fourth Military Medical University, Xi’an, Shaanxi, China; 2 Department of Orthopedics, Xijing Hospital, Fourth Military Medical University, Xi’an, Shaanxi, China; 3 Department of Burns and Cutaneous Surgery, Xijing Hospital, Fourth Military Medical University, Xi’an, Shaanxi, China

**Keywords:** osteosarcoma, Na^+^/H^+^ exchanger 1, reactive oxygen species, proliferation, migration

## Abstract

**Background:**

Osteosarcoma (OS) is the most common primary malignant bone tumor in children and adolescents. OS is associated with locally aggressive growth and high metastatic potential. The mechanisms that underlie these processes are currently elusive. Reactive oxygen species (ROS) and Na^+^/H^+^ exchanger 1 (NHE1) have been suggested to regulate proliferation and migration of tumor cells. However, the relationship between NHE1 and ROS in OS proliferation and migration has not been investigated before.

**Aim:**

To investigate the role of NHE1 and ROS in the proliferation and migration of OS.

**Methods:**

ROS levels and NHE1 expression were studied in cultured human OS cells and human OS xenografts in nude mice. In vitro, OS cells were treated with different doses of tert-butyl hydroperoxide (tBHP), a ROS inducer, and cariporide, an NHE1 inhibitor, to study the effect on cell proliferation and migration. In vivo, nude mice bearing OS cells were administrated with NHE1 inhibitor or antioxidant and the tumor weights were measured.

**Results:**

This study reported for the first time that the expression of NHE1 and intracellular ROS level were both increased in OS tissues and cells. Exposure of OS cell to ROS derived from tBHP was able to accelerate cell proliferation and migration and also up-regulate NHE1 protein expression. Moreover, tBHP significantly increased intracellular pH (pHi), decreased extracellular pH (pHe) and induced upregulation of ERK, MMP2, and MMP9. Lowering of ROS levels with the anti-oxidant DMTU or inhibiting NHE1 activity via cariporide abolished the stimulatory effect of tBHP. However, there cariporide did not affect intracellular ROS levels. In vivo study we further confirmed that cariporide could inhibit tumor growth in the nude mouse xenografts of OS cells.

**Conclusions:**

The data demonstrate that up-regulation of NHE1 was induced by low concentrations of ROS contributes to the regulation of tumor proliferation and invasion of OS.

**Relevance for patients:**

There is potential application for cariporide as an effective antitumor agent during the development of human osteosarcoma. In addition, redox modulation on proton transport may represent a novel target of osteosarcoma prevention, and open a new avenues for future research.

## 1. Introduction

Osteosarcoma (OS) is the most common primary malignant bone tumor in children and adolescents. OS is associated with locally aggressive growth and an early stage of metastatic potential [1]. The prognosis in patients with metastasis or recurrence is bleak, as reflected by a 5-y survival rate of 20% [2]. Accordingly, there is a medical need to improve the clinical management of OS by e.g., the development of novel therapeutics to deter cancer progression and metastasis and improve the patients’ survival rates. An understanding of the molecular mechanisms that drive OS progression and metastasis will help in the identification of new therapeutic intervention sites.

Among the many factors involved in the maintenance of homeostatic cell growth is intracellular pH (pHi), which is tightly regulated in tumor cells. Studies have revealed that tumor cells maintain an alkaline pHi and that the pH in the tumor stroma (extracellular pH, or pHe) is acidic [3]. Elevation of the pHi is believed to promote cell proliferation while a reduction in pHe is involved in the early stage of cancer cell migration, invasion, and metastasis [4,5]. The sodium/hydrogen antiporter Na^+^/H^+^ exchanger 1 (NHE1) plays a key role in the regulation of pHi. NHE1 has also been directly linked to cell transformation, invasion, and metastasis [6-8]. Accordingly, NHE1 is a potentially druggable target for anti-cancer therapeutics.

Some of the aforementioned biological processes modulated in part by NHE1 are also regulated by the intracellular redox state. For example, exogenously administered reactive oxygen species (ROS) stimulate growth and growth responses in a variety of cultured mammalian cell types [9]. An increase in intracellular ROS levels leads to several key alterations in cancer cells, including cell proliferation and transformation [10]. Correspondingly, the malignant phenotype of cancer cells can be reversed by decreasing intracellular ROS levels [11,12]. Inasmuch as ROS also modulate pHi by inhibiting [13,14] or activating [15] NHEs, the interplay between intracellular ROS and NHE1 may be a regulatory mechanism underlying tumor cell proliferation and invasion.

Currently, little is known about the roles of ROS and NHE1 in proliferation and migration of OS cells. We therefore tested the hypothesis that ROS-mediated activation of NHE1 plays a role in OS cell proliferation and migration. First, elevated NHE1 protein and hydrogen peroxide (H_2_O_2_) levels were confirmed in tissue biopsies of OS patients to underpin their potential involvement in the tumor biology of clinical OS. Next, a link between ROS and cell proliferation and NHE-1 modulation was established in cultured OS (U2OS) cells in several experimental test arms focused on cell proliferation and migration. The experiments revealed that (1) ROS promotes OS cell proliferation and activates NHE1, (2) inhibition of NHE1 decreases ROS-mediated OS cell proliferation, and (3) inhibition of NHE1 decreases ROS-mediated migration of OS cells. Finally, the in vitro findings were validated in a murine xenograft model of human OS, confirming that NHE1 inhibition by cariporide, a specific NHE1 inhibitor, leads to suppressed tumor growth. Accordingly, pharmacological inhibition of NHE1 may aid in the clinical management of OS.

## 2. Materials and methods

### 2.1. Patient samples

The use of tissue biopsies from OS patients who had undergone limb salvage or amputation surgery was approved by the institutional review board of the Xijing Hospital, Fourth Military Medical University, under protocol number KY2015-0120-3. The relevant medical information of the 24 included patients was retrieved from the patients’ health records and is presented in Table 1. The metastasis-incompetent or metastasis-competent classification of OS was based on the clinical diagnosis (x-ray, chest CT, and biopsy). Biopsies were collected at the time of diagnosis, before preoperative chemotherapy, near the tumor-bone interface. Non-malignant bone tissue was collected from 5 patients who had undergone hip or knee replacement surgery.

**Table 1.** Medical details of the osteosarcoma patients from which tissue biopsies were derived for the determination of NHE1 protein and hydrogen peroxide levels. The osteosarcomas were classified by their phenotype, namely metastasis-incompetent tumors (MI) and metastasis-competent tumors (MC).

Variable		Patient biopsies, N (%)	
		MI	MC
*Gender*			
	Male	4 (17)	9 (38)
	Female	5 (21)	6 (25)
*Age*			
	Median	13.5	15.5
	Range	11-25	10-19
*Tumor site*			
	Femur	5 (21)	8 (33)
	Tibia	2 (8)	5 (21)
	Humerus	1 (4)	0
	Pelvis	0	1 (4)
	Other	1 (4)	1 (4)

The biopsies were acquired during standard patient work-up and were therefore retrieved from the tissue bank and retrospectively processed for immunohistochemical analysis (section 2.2). Because the samples were obtained as part of standard medical procedures and because the data cannot be traced back to the patients in any way, the institutional review board provided an exemption for acquiring informed consent.

### 2.2. Immunohistochemistry

Tissue biopsies were fixed in 10% buffered formalin, dehydrated in graded steps of ethanol and xylene, and embedded in paraffin. The samples were subsequently cut into 5-μm thick sections. Following deparaffinization, endogenous peroxidase activity was blocked by incubating the sections with 3% H_2_O_2_, after which the sections were washed with water. The sections were incubated with goat serum for 10 min at room temperature, followed by overnight incubation with rabbit polyclonal anti-NHE1 antibody (Santa Cruz Biotechnology, Santa Cruz, CA, 1:200 dilution) at 4 °C. Next, sections were incubated with biotinylated goat anti-rabbit IgG secondary antibody (Jinshan, Beijing, China) according to the manufacturer’s instructions. The labeled antigens were revealed by staining with 3,3'-diaminobenzidine (DAB, Sigma-Aldrich, St. Louis, MO).

The sections were imaged by light microscopy (Olympus BX51, Tokyo, Japan) and analyzed for NHE1 staining intensity using Image-Pro Plus (Media Cybernetics, Rockville, MD). Five sections were analyzed per biopsy, whereby the optical density of DAB was quantified in three randomly selected high-power fields (200× magnification).

### 2.3. Cell culture and treatment

The human OS cell lines U2OS, HOS, HOS-143B, and KHOS as well as the osteoblast cell line hFOB 1.19 were purchased from American Type Culture Collection (Rockville, MD). The cells were cultured in DMEM medium (Gibco/Life Technologies, Carlsbad, CA) supplemented with 10% fetal bovine serum (Gibco/Life Technologies). Cells were grown under standard culture conditions (37 °C, humidified atmosphere composed of 95% air and 5% CO_2_).

U2OS cells were incubated with tert-butyl hydroperoxide (tBHP, 1–100 μM, Sigma-Aldrich), the antioxidant 1,3-dimethyl-2-thiourea (DMTU, 30 mM, Sigma-Aldrich), or the specific NHE1 inhibitor cariporide (10 μM, Sigma-Aldrich) that had been added to serum-free medium directly before incubation with cells. Cariporide was dissolved in dimethyl sulfoxide (DMSO, final concentration of 0.1%).

For the ROS and Western blot assays, the cells were harvested after 4 h incubation. For the cell viability assays, cells were harvested at the indicated time points after incubation. For cell cycle and apoptosis assays, cells were harvested after 24 h incubation.

### 2.4. Reactive oxygen species quantification in osteosarcoma tissue biopsies and cultured cells

Tissue biopsies were immediately snap frozen in liquid nitrogen and stored at –80 °C until further use. The frozen tissue samples were weighed and placed in 1 mL ice-cold buffer (50 mM potassium phosphate buffer, pH = 7.4, containing 0.48 M sodium azide). Next, 20% (w/v) tissue homogenates were prepared with a Teflon homogenizer. The crude homogenates were centrifuged at 16,000 ×g for 10 min. The supernatants were collected for H_2_O_2_ determination.

The H_2_O_2_ concentration in total liver homogenates was determined using an Amplex Red Hydrogen Peroxide Assay kit (Thermo Fisher Scientific, Waltham, MA) according to the manufacturer's protocol. Standard solutions with a known H_2_O_2_ concentration were prepared to construct a standard curve. The standard solutions were diluted with the same buffer as used for the preparation of tissue homogenates. Fluorescence emission was measured at λ_ex_ = 545 nm and λ_em_ = 585 nm on a Tecan Infinite 200 multi-mode microplate reader (Tecan, Männedorf, Switzerland). The data were expressed as the mean ± SD H_2_O_2_ concentration (absolute values in μM).

Intracellular ROS were analyzed by flow cytometry following cell labeling with the fluorogenic redox probe 2′,7′-dichlorodihydrofluorescein diacetate (DCFH_2_-DA; Sigma-Aldrich). DCFH_2_-DA is a cell-permeable, non-fluorescent probe that enters the cell. Intracellularly, the diacetate moieties are cleaved by cellular esterases, forming the non-fluorescent DCFH_2_. DCFH_2_ is oxidized by ROS to the highly fluorescent DCF. The DCF fluorescence intensity is stoichiometrically proportional to the amount of intracellular ROS formed.

Briefly, cells were seeded into 6-well plates for 24 h and, where applicable, treated with the indicated reagents for 4 h, followed by incubation with 10 μM DCFH_2_-DA in PBS for 30 min at standard culture conditions. The cells were detached by trypsinization and centrifuged at 100 ×g for 5 min. The supernatant was removed and the cells were resuspended in PBS. DCF fluorescence was measured at λ_ex_ = 480 nm and λ_em_ = 525 nm using a FACSCalibur flow cytometer (BD Biosciences, Franklin Lakes, NJ). Data (mean FL1 fluorescence intensity) were analyzed using Accuri C6 software (BD Biosciences). The fluorescence intensities were corrected for the autofluorescence of cells that had not been incubated with DCFH_2_-DA. ROS levels were expressed as percentage of control.

### 2.5. Cell viability assay

Cell viability was determined using a commercial WST-1 kit (Beyotime, Haimen, China). Briefly, U2OS cells were seeded at a density of 5×10^4^ cells/cm^2^ in 96-well plates. Cells were allowed to attach for 12 h under standard culture conditions and incubated with increasing tBHP concentrations for 0, 4, 8, or 24 h. Following incubation, the medium (200 μL) was refreshed and 10 μL of WST-1 reagent was added to each well. The cells were cultured for 1 h at standard conditions. Finally, the absorbance was read at 450 nm in a microplate reader (Tecan Sunrise, Männedorf, Switzerland).

### 2.6. Cell cycle and cell death analysis

Cultured U2OS cells that had been subjected to 24 h treatment (section 2.3) were fixed overnight in 70% ethanol at 4 °C. After detachment and centrifugation, cells were stained with 0.1 μg/mL RNase A (Beyotime) for 30 min at 37 °C and subsequently with 50 μg/mL propidium iodide (PI, Beyotime) for 30 min at 4 °C. Next, the cells were assayed by flow cytometry (EPICS XL, Beckman Coulter, Brea, CA) at λ_ex_ = 488 nm and emission in the FL3 channel. Each histogram was constructed on the basis of at least 5,000 events. Data were analyzed for cell cycle phases by calculating the percentage of cells in each phase using multicycle DNA cell cycle analysis software (FACScan, BD Biosciences).

Apoptosis was determined with an annexin V-FITC apoptosis detection kit (Beyotime) according to the manufacturer’s instructions. Briefly, U2OS cells were seeded into 6-well plates for 24 h and treated with the indicated chemical reagents for 24 h. After incubation the cells were collected, washed with PBS, resuspended in annexin V binding buffer, and incubated with annexin V-FITC/PI in the dark for 15 min. The cells were assayed by flow cytometry (EPICS XL, Beckman Coulter) at λ_ex_ = 488 nm and emission in the FL1 (FITC) and FL3 (PI) channels. The extent of apoptosis (propidium iodidepositive and annexin V-positive cells) was analyzed with FACS software (Beckman).

### 2.7. Measurement of pHi and pHe

The pHi was measured in U2OS cells using the pH-sensitive fluorophore 2',7'-bis-(2-carboxyethyl)-5-(and-6)-carboxyfluorescein, acetoxymethyl ester (BCECF-AM, Invitrogen/ Life Technologies) as described in [16]. The cells were incubated with 1 μM BCECF-AM (final concentration in medium) for 30 min and read in a luminescence plate reader (Tecan Sunrise) at λ_ex_ = 490 and 450 nm and an emission wavelength of 535 ± 10 nm. The fluorescence emission intensity ratio (F490/F450) was calculated and the pHi was derived on the basis of the high-K^+^-nigericin method [17].

The pHe was measured spectrophotometrically using phenol red as pH indicator as described previously [18]. The ratio of 450/490 nm absorbance was converted to pH using the following equation: pH = log((R-R_min_) / (R_max_-R)) + pKa, where R is the experimentally derived ratio of absorbance, R_max_ and R_min_ indicate the boundary values of R, and pKa indicates the negative log of the dissociation constant (pK_a_ = 7.5 for phenol red). R_max_ and R_min_ values were calculated from a standard curve for each experiment.

### 2.8. Western blot analysis

Patient-derived tissue biopsies and harvested cells were lysed in RIPA buffer (Beyotime) for 30 min on ice. The lysate was centrifuged at 20,000 ×g for 20 min at 4 °C. The supernatant was transferred, and protein concentration was determined using the BCA Protein Assay kit (Thermo Fisher Scientific). Equal amounts of total and nuclear proteins were separated by sodium dodecylsulfate-polyacrylamide gel electrophoresis and electro-transferred onto a nitrocellulose membrane. Membranes were incubated with antibodies to NHE1 (Santa Cruz Biotechnology), CyclinD1 (Abcam, Cambridge, UK), PCNA (Abcam), MMP-2 (Santa-Cruz), MMP-9 (Santa Cruz Biotechnology), ERK1/2 (Epitomics, Burlingame, CA), phospho-ERK1/2 (Epitomics). The secondary antibodies used for detection were HRP-conjugated anti-rabbit and anti-mouse IgG (Santa Cruz Biotechnology). Immunoreactive bands were detected by an enhanced chemiluminescence kit (EMD Millipore, Darmstadt, Germany) and results were quantitated using an image analyzer Quantity One System (Bio-Rad Laboratories, Hercules, CA). β-actin and LaminB (Santa Cruz Biotechnology) were used as loading control for total and nuclear fractions, respectively.

### 2.9. Wound healing assay

Equal numbers of U2OS cells were seeded in 6-well plates and grown under standard conditions. At 90% confluence, a single wound was created by gently scraping the attached cells using a sterile plastic pipette tip. Debris was removed by washing the cells with serum-free medium, and serum-free medium containing reagents (section 2.3) was added to the cells. Cell migration of the cells into the wounded area was recorded 24 h after wound induction. The migratory patterns and extended protrusion of cells from the border of the wound were visualized and photo-documented using an inverted microscope (Olympus CKX41, Tokyo, Japan). Uncovered areas in the wound were quantified as the percentage of the original wound area.

### 2.10. In vitro invasion assay

Cell migration and invasion were determined with Matrigel-coated Transwell cell culture chambers (8-μm pore size) (EMD Millipore) as described previously [19]. U2OS cells were seeded (1×10^4^ cells/well) into the upper chamber with serum-free medium. The bottom chamber contained medium with 0.5% DMSO, cariporide, or DMTU + cariporide. After 48 h of incubation, the cells in the upper chamber were removed and the cells that invaded through the membrane were fixed with 70% ethanol and stained with 2% crystal violet in 2% ethanol. Invaded cells in six random microscopic fields (200 × magnification) were counted and all experiments were performed in triplicate.

### 2.11. In vivo tumorigenesis studies

All animal experimentation was approved by the institute’s ethics committee (protocol #15009). Male 6-week-old BALB/c nude mice (N = 18) were obtained from the Experimental Animal Research Center at the Fourth Military Medical University and treated in accordance with the Guide for the Care and Use of Laboratory Animals (NIH publication 85-23, rev. 2011) during the entire experiment. Animals were housed under barrier conditions in a climate-controlled chamber with 12 h light/dark cycles and ad libitum access to sterilized water and chow. The mice were acclimated for 7 d before entering the experiment.

For tumor xenografting, 3×10^6^ 143B cells were injected subcutaneously at the level of the femur and the animals were randomly divided into three groups (N = 6 per group). Cariporide and DMTU were administered intraperitoneally in sterile isotonic saline solution at 5 and 500 mg/kg (200 μL injection volume), respectively, twice a day for 8 wk. The control group received 200 μL of PBS. At the end of the experiments, mice anesthetized by intraperitoneal injection of 150 mg/kg pentobarbital and sacrificed by cervical dislocation, the tumors were excised, and the tumor weights were measured.

### 2.12. Statistical analysis

Statistical analysis was performed in GraphPad Prism (GraphPad Software, San Diego, CA). Normally distributed data sets (determined with a Kolmogorov-Smirnov test) were compared using a one-way ANOVA followed by Dunnet's post-hoc test where applicable. A *P*-value of ≤ 0.05 was considered statistically significant. Data were expressed as mean ± SD.

## 3. Results

### 3.1. NHE1 expression in patient-derived osteosarcoma tissue and cultured osteosarcoma cells

**Figure 1.**
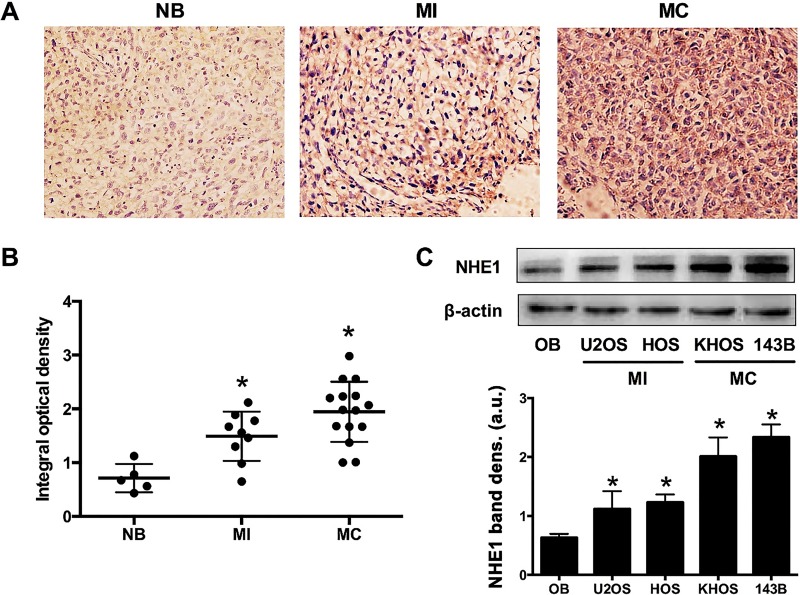
Detection of Na^+^/H^+^ exchanger 1 (NHE1) expression in patient-derived osteosarcoma (OS) tissue and cultured OS cells. (A) Immunohistochemical staining of NHE1 protein in biopsies of non-malignant bone (NB), metastasis-incompetent OS (MI), and metastasis-competent OS (MC). The antigens were revealed by DAB staining, yielding a brown color in NHE1-positive tissue. Magnification ×200. (B) Quantitative assessment of NHE1 protein expression levels in immunohistochemically stained tissue sections confirmed the observational findings and revealed significantly higher DAB staining in MI and MC OS tissue compared to NB tissue. (C) Western blot analysis of NHE1 expression in different OS cell lines and nonmalignant osteoblast (OB) cell line. Representative data from three independent experiments are shown. The NHE1 band intensity was normalized to the band intensity of β-actin. *P < 0.05 compared to OB.

Differences in NHE1 protein expression in normal tissue biopsies (non-malignant bone (NB); N = 5), primary tumor biopsies from metastasis-competent (MC) OS (N = 14), and from metastasis-incompetent (MI) OS (N = 9) were determined by immunohistochemistry and Western blot. NHE1 protein was mainly present in the cell membrane, exhibiting a heterogeneous distribution pattern. Positive staining for NHE1 protein was observed in 66.7% (6/9) of MI biopsies and in 71.4% (10/14) of MC biopsies, whereas only 20% (1/5) of the NB biopsies exhibited NHE1 positivity (Figure 1A). In terms of staining intensity, MI and MC OS biopsies had significantly higher expression of NHE1 (*P* = 0.029 and *P* = 0.0087, respectively) than NB (Figure 1B).

To confirm elevated NHE1 protein levels in cultured OS cells for subsequent in vitro experiments, NHE1 expression was evaluated by Western blot in four different OS cell lines by western blotting. Human osteoblast (OB) cells were used as a reference. U2OS and HOS cells belong to MI category, while KHOS and 143B cells belong to MC category. NHE1 expression levels were significantly higher in all four OS cell lines compared to OB cells (Figure 1C). Taken together, the in vitro OS model system bears analogy to the in vivo situation in terms of NHE1 protein expression.

### 3.2. Reactive oxygen species levels in patient-derived osteosarcoma tissue and cultured osteosarcoma cells

**Figure 2.**
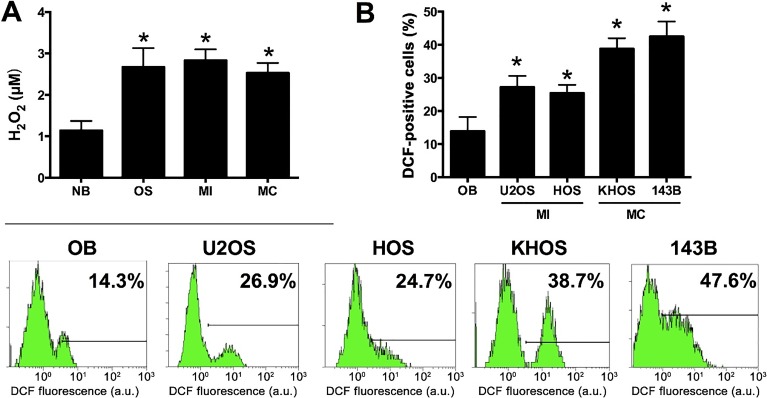
ROS levels in patient-derived non-malignant bone (NB, N = 5) and osteosarcoma (OS, N = 23) tissue (A) and cultured osteosarcoma cells (B). In (A), H_2_O_2_ was quantified using an Amplex Red assay on tissue homogenates. Data were also stratified into metastasis-incompetent (MI, N = 9) and metastasis-competent (MC, N = 14) OS. *P < 0.05 compared to the NB group. (B) Different OS cell lines and a non-malignant osteoblast (OB) cell line were incubated with DCFH_2_-DA and analyzed by flow cytometry to determine the levels of intracellular ROS. Data represent mean ± SD of N = 5. *P < 0.05 compared to the OB group.

Because we hypothesized that ROS play a role in the NHE1 signaling axis, ROS levels were determined in patient derived OS tissue and cultured cells and compared to their non-malignant counterparts. As shown in Figure 2A, the H_2_O_2_ concentration in OS (MI and MC clustered), MI OS, and MC OS tissue was significantly higher than that in NB tissue. Similarly, ROS levels were also significantly higher in all four OS cell lines compared to OB cells (Figure 2B). Collectively these results indicate that increased NHE1 expression and intracellular ROS levels are pronounced molecular signatures in primary OS.

To test the interconnectedness between ROS and NHE1, we next determined whether an increase in ROS levels would be accompanied by an increase in NHE1 expression, which may mechanistically underlie the malignant transformation of normal bone into OS.

### 3.3. Reactive oxygen species promote osteosarcoma cell proliferation and dose-dependently activates NHE1

**Figure 3.**
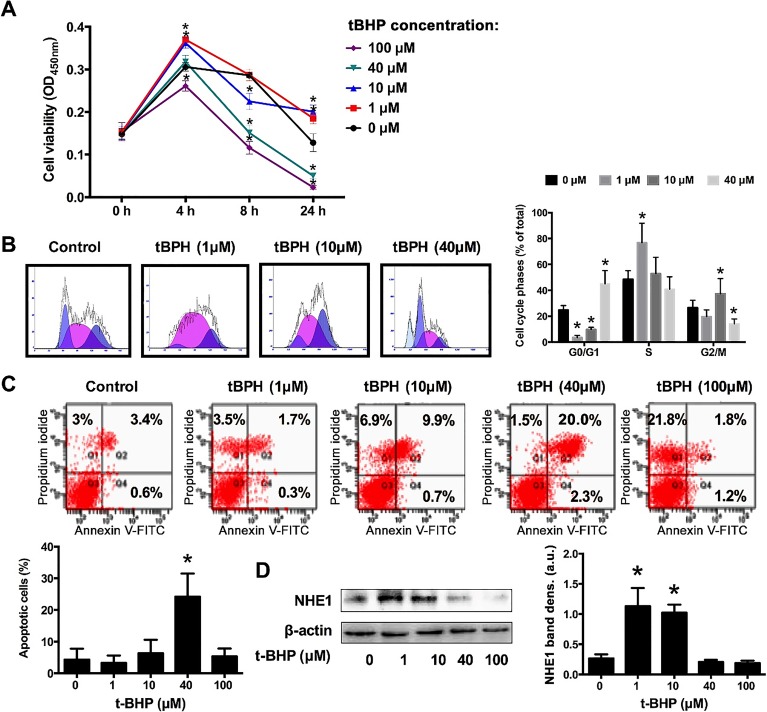
ROS induces proliferation in U2OS cells and activates NHE1 dose-dependently. Cell viability (A), cell cycle (B), apoptosis (C), and NHE1 protein expression (D) were analyzed after incubation with increasing concentrations of the ROS precursor tBHP. In the flow cytograms of (C), a cell population in the upper right corner (PI- and annexin V-positive) is characteristic of late apoptotic cells, whereas a cell population in the lower right corner (PI-negative and annexin V-positive) typifies early apoptotic cells. The quantification was performed on right upper and lower quadrant cells. Data represent mean ± SD of N = 5. *P < 0.05 compared to control.

To investigate the role of ROS in OS cell proliferation, U2OS cells were incubated with increasing concentrations of tBHP (1, 10, 40, and 100 μM), which is a directly-acting ROS-generating agent, for 4, 8, or and 24 h. As shown in Figure 3A, the viability of cells treated with 1-10 μM tBHP, corresponding to a mild degree of ROS hyperproduction, for 4 h and 24 h was significantly higher compared to control cells (0 μM tBHP).

Cell cycle analysis was performed to confirm that the increase in cell viability under mild ROS-producing conditions was indeed due to proliferative signaling. Cells treated with 1 μM tBHP for 24 h exhibited augmented S phase activity (i.e., DNA replication) and reduced G1 phase activity (Figure 3B), confirming the proliferative state of these cells under mild ROS-producing conditions.

Apoptosis was also evaluated after incubation with tBHP for 4 h to examine tBHP induced-cytotoxicity. As shown in Figure 3C, U2OS cells underwent early apoptosis after incubation with 40 μM tBHP, whereas cells exposed to 100 μM tBHP were mainly late apoptotic. A turning point between early and late apoptosis was observed at 10 μM tBHP (Figure 3C, flow cytogram), whereas the 1-μM tBHP concentration was largely non-toxic to cells.

### 3.4. Low levels of reactive oxygen species activate NHE1

We further examined the effect of tBHP on the protein expression of NHE1 in U2OS cells. As shown in Figure 3D, NHE1 expression was strongly upregulated in response to 1-10 μM tBHP. There was no effect on NHE1 expression at much higher tBHP concentrations (40-100 μM). These data provide compelling evidence that NHE1 is redox-regulated under mild pro-oxidative conditions.

On the basis of the results in Figure 3, a fixed non-toxic concentration of tBHP (1 μM) was used in subsequent experiments.

### 3.5. Inhibition of NHE1 decreases reactive oxygen species-induced proliferation of osteosarcoma cells

To address the biochemical interconnectedness of ROS and NHE1 in tBHP-induced cell proliferation, we used the anti-oxidant DMTU to reduce ROS levels and cariporide to selectively inhibit the activity of NHE1. The DMTU and cariporide concentrations employed here were based on preliminary experiments in U2OS cells that ruled out notable effects of the used compound concentrations on the studied parameters (data not shown).

**Figure 4.**
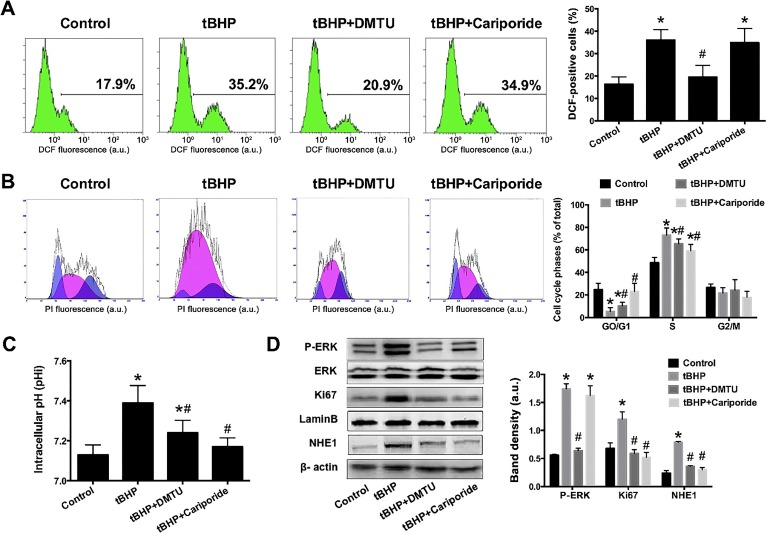
Inhibition of NHE1 decreased ROS-induced OS cell proliferation. Intracellular ROS (A), cell cycle (B), pHi (C), and immunoblots of pERK, Ki67, and NHE1 (D) were assayed after treating U2OS cells with 1 μM tBHP alone or in combination with DMTU or cariporide. Data represent mean ± SD of N = 5. *P < 0.05 versus control. ^#^P < 0.05 versus the tBHP group.

As shown in Figure 4A, a significant increase in DCF fluorescence was observed in U2OS cells after 4 h incubation with 1 μM tBHP, confirming the ROS-generating potential of tBHP. DMTU treatment markedly reduced intracellular ROS levels in the presence of tBHP. Cariporide did not exert a ROS-ameliorating effect.

Moreover, we observed that both DMTU and cariporide were similarly efficient in reducing the degree of S phase activity (Figure 4B). The cariporide-mediated decrease in S phase activity, which did not affect the extent of ROS production (Figure 4A), suggests that cell proliferation is not solely governed by intracellular redox states but also by NHE1-related phenomena, e.g., pHi.

We therefore examined the effect of tBHP on pHi values in U2OS cells. As shown in Figure 4C, the pHi values in tBHP-treated cells were significantly higher than those in untreated control cells. DMTU or cariporide both reduced the pHi, suggesting that OS cell proliferation is regulated by intracellular redox state and pHi, whereby the latter may be modulated by NHE1.

To investigate the relationship between NHE1 and proliferation more closely, key molecular regulators of proliferation and NHE1 were assayed by proteomics as a function of the intervention. Ki67 is a nuclear protein that is associated with cell proliferation. As shown in Figure 4D, Ki67 was upregulated by tBHP and this effect was abolished by DMTU and cariporide treatment, which is consistent with the cell cycle analysis (Figure 4B). ERKs are widely expressed protein kinases that are involved in the regulation of proliferation (i.e., meiosis, mitosis, and post-mitotic processes) in differentiated cells upon activation by phosphorylation. We speculated that DMTU would revert the expression of EKR and NHE1 that was induced by tBHP treatment. Indeed, mild intracellular ROS production was associated with increased levels of phospho-ERK, which was abrogated by DMTU but not cariporide (Figure 4D). Both DMTU and cariporide reversed the upregulation of NHE1 by tBHP.

These results demonstrated that ROS and NHE1 are involved in tBHP-induced U2OS cell proliferation, but by different mechanistic pathways. ROS mainly signal through the ERK pathway, whereas NHE1 may regulate OS cell proliferation through pHi modulation. However, ROS are responsible for NHE1 activation - a step that seems to be important for proliferative signaling via changes in pHi.

### 3.6. Inhibition of NHE1 decreases reactive oxygen species-induced migration of osteosarcoma cells

**Figure 5.**
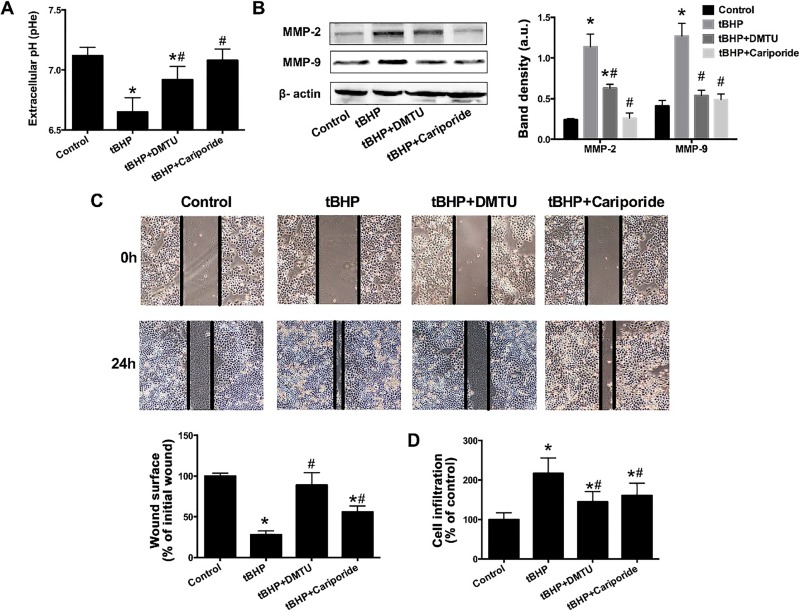
Inhibition of NHE1 decreased the ROS-induced migration of OS cells. pHe (A), immunoblots of MMP-2 and MMP-9 (B), wound healing ability (C), and invasion (D) were analyzed after treating U2OS cells with 1 μM tBHP alone or in combination with DMTU or cariporide. Data represent mean ± SD of N = 5. *P < 0.05 versus control. ^#^P < 0.05 versus the tBHP group.

We then sought to determine whether and how ROS affected the invasion and migration properties of U2OS cells. First, the effect of tBHP on the pHe was investigated, given its role in the early stage of cancer cell migration, invasion, and metastasis [4,5]. As shown in Figure 5A, 1 μM tBHP significantly lowered the pHe in U2OS cell cultures versus the control group. Treatment with DMTU or cariporide considerably reversed pHe levels that were reduced by tBHP, indicating that mild oxidative stress and NHE1 also play a role in pHe regulation.

An acidic pHe is believed to be critical for the activation of extracellular proteases [38,39]. Accordingly, the expression of matrix metalloproteinases (MMPs), which are proteases that degrade the extracellular matrix during e.g., metastasis, was determined. The results evinced that MMP-2 and MMP-9 were significantly upregulated by tBHP. DMTU and cariporide treatment reversed the tBHP-induced expression of MMP-2 and MMP-9 (Figure 5B).

Cell migration was measured by means of the wound healing assay 24 h after wound induction (Figure 5C). Compared to control, tBHP promoted the migration of U2OS cells, an effect that was abrogated by DMTU and moderately inhibited by cariporide. The results were confirmed by using a Matrigel-coated transwell cell culture chambers. In agreement with the wound healing data, the migration assay showed that there is a significant promotive effect of tBHP on cell migration (Figure 5D), whereby co-incubation with DMTU or cariporide for 24 h significantly reduced the tBHP-induced cell migration. These data attest that the ROS-induced pHe decrease and cell migration were mediated by NHE1, and that MMPs were probably involved in this process.

### 3.7. Cariporide suppresses tumor growth in nude mice bearing osteosarcoma xenografts

**Figure 6.**
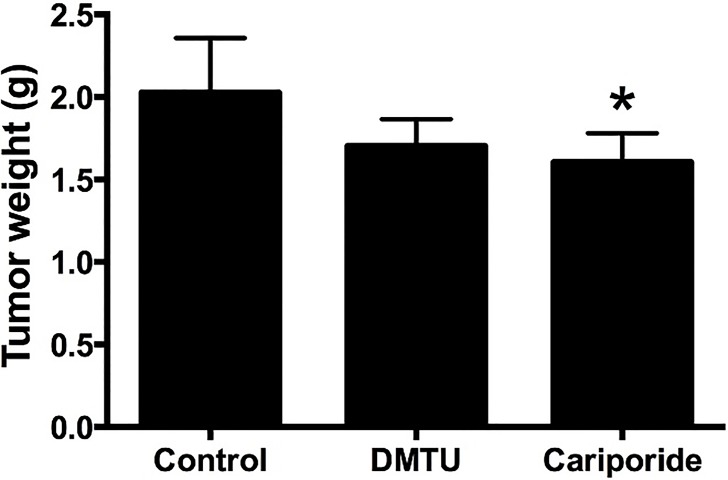
Cariporide-mediated suppression of tumor growth in the nude mice bearing human OS xenografts. Data represent mean ± SD of N = 6. *P < 0.05 versus control.

In the final experimental test arm a mouse human OS xenograft model was used to translate the in vitro effects of DMTU and cariporide to an in vivo situation. As shown in Figure 6, tumor growth was significantly inhibited at a dose of 0.5 mg/kg cariporide compared to the control group (*P* = 0.0203). The tumor weight in control- and DMTU-treated animals did not differ (*P* = 0.0576).

## 4. Discussion

This study revealed that patient-derived OS and cultured OS cells exhibit increased levels of ROS production and greater expression of NHE1 compared to non-malignant bone tissue and non-OS cells. In focused in vitro experiments it was shown that cell proliferation and migration are governed by ROS and NHE1, albeit by different mechanisms. ROS induce cell proliferation directly through ERK and indirectly through NHE1 by augmenting the pHi and downmodulating the pHe. How shifts in pH in the different compartments of OS cells signal proliferation and migration is currently elusive, al-though the migration of OS cells possibly involves MMP-2 and MMP-9 that in turn are activated by pHe [38,39]. Furthermore, it was shown that DMTU (anti-oxidant) and cariporide (NHE1 inhibitor) confer anti-proliferation and anti-migration effects in cells. The suppressive effect of cariporide on tumor growth was validated in nude mice bearing human OS xenografts. Collectively, the results revealed that NHE1 mediates ROS-induced cell proliferation and migration and that inhibition of NHE1 by cariporide comprises an effective intervention in terms these processes.

There is increasing evidence indicating that an important survival strategy of tumor cells in a hostile microenvironment is to deter intracellular acidification that might lead to apoptotic cell death [20]. In this context, one of the primary and best-studied regulators of both pHi and pHe in tumors is NHE. Elevated NHE activity is correlated with an increase in pHi and a decrease in pHe, whereby the proton shift is associated with the initiation and local growth of tumors as well as metastasis [21]. Overexpression of NHE1 has repeatedly been shown to contribute significantly to the phenotype of many types of cancer cells [21]. Our previous studies have shown that the expression level of NHE1 is directly correlated with tumor progression and inversely related to prognosis in hepatocellular carcinoma, and that NHE1 could be a potential therapeutic target for hepatocellular carcinoma [22,23]. However, there are no reports yet for the role of NHE1 in OS.

OS tend to appear at the sites of bone growth, presumably because proliferation makes osteoblastic cells in this region prone to mutations that could lead to cell transformation [24]. It has been proven that an actively maintained pH gradient is important for normal osteoblasts function [25,26]. In light of these findings, we speculated that dysregulation of the normophysiological pH balance is involved in OS development. We compared NHE1 protein expression patterns between human-derived normal and OS tissues and cells. The results showed that NHE1 was overexpressed in both, and that NHE1 overexpression was indeed associated with a shift in pHi/pHe. However, the mechanisms by which NHE1 is activated in tumor cells have been controversial, and are still not completely elucidated.

Cancer cells typically have higher levels of ROS than normal cells [11,12]. Our study showed that ROS levels were higher in OS tissues and cells compared to their non-malignant counterparts. Numerous studies demonstrated that low levels of ROS stimulate in vitro growth of many cell types, while higher levels of ROS can induce cell death [27], reflecting a biphasic effect of ROS on cell growth. The biphasic character of ROS was also evident in OS cells, as clearly demonstrated in this study. At 1-10 μM tBHP, a hydrogen peroxide precursor, the OS cells exhibited a pro-proliferative phenotype, while at higher levels (> 40 μM) of tBHP the cells underwent programmed cell death. Previous studies found that ROS can activate or inhibit NHEs, but the link between ROS levels and NHE levels was not well defined. Our results showed that ROS also induced a biphasic effect on NHE1, where 1-10 μM tBHP strongly upregulated NHE1 protein expression. This effect was reverted at higher ROS concentrations. Furthermore, the biological functions of ROS are widespread, but an important function is the inhibition of protein tyrosine phosphatases (PTPs) through stepwise oxidation of a critical cysteine residue at the catalytic site [28]. PTPs make up a large family of enzymes involved in many cellular signaling pathways. A recent study suggested that PTPs also affected the regulation of pH by physically interacting with NHE [29]. These mechanisms may explain some of the regulatory effects of ROS on NHE1 in our study.

Early experiments have demonstrated that ROS-mediated cell proliferation is in part governed by a shifting pHi [30] and that migration is facilitated by an acidic pHe [31], which in turn activates MMPs to accommodate the migration process through extracellular matrix remodeling [32]. Corroboratively, our data showed that low levels of ROS increased the pHi and decreased the pHe, underscoring the redox dependence of these processes. We therefore investigated whether a ROS-mediated change in NHE1 expression is a possible mechanism underlying pH-mediated cell proliferation and migration.

It has been demonstrated that the treatment of various types of cancer cells with selective and potent inhibitors of NHE1, including cariporide, suppresses their invasive propensity [33]. However, how to therapeutically intervene in NHE1-mediated cell processes with cariporide is yet to be determined [34]. The utilization of this drug in cancer treatment is currently underexplored and there is limited experimental data on NHE1 up-regulation in tumor cells [35]. Our results showed that cariporide abolished the ROS-induced proliferation of OS cells through its selective inhibition of NHE1 and subsequent decrease in pHi. The cell cycle phases and Ki67 were also favorably affected by cariporide in terms of anti-cancer pharmacology, which was experimentally corroborated in the murine human OS xenograft model. However, ROS activation of ERK was NHE1-independent, which is consistent with previous findings that ERK acts upstream of NHE1 [36]. Antioxidant treatment of mice did not lead reduced tumor size, although this may have been attributable to reasons related to intraperitoneal DMTU pharmacokinetics and disposition rather than amelioration of intratumoral ROS levels per se.

In our study, cariporide also inhibited the ROS-induced migration of OS cells that concurred with an increased pHe. The mechanisms responsible for NHE1-mediated tumor metastasis are complex, involving a number of biochemical and cellular events. MMPs play a key role in degradation of the extracellular matrix, leading to an ability of tumor cells to invade tissue during metastasis [37]. Because acidic pHe has been shown to result in MMP upregulation [38,39], we hypothesized that these metastasis-facilitating proteins are involved in NHE1-mediated invasion. Cariporide treatment, and hence inhibition of NHE1, reduced MMP-2 and MMP-9 expression, which suggests that the NHE1-mediated invasive behavior of OS cells coincides with upregulation of MMP-2 and MMP-9 via NHE1. We acknowledge that the exact mechanism by which NHE1 facilitates OS cell invasion requires more detailed investigation. NHE1-mediated acidification of the extracellular milieu and activation of MMPs may be part of the mechanism [38,39] and should therefore be studied more closely.

In conclusion, our study demonstrated increased ROS production and NHE1 expression in OS tissues and cell lines. Inhibition of ROS or NHE1 activity reduces OS cell proliferation and invasion. Moreover, NHE1 levels are redox-regulated and steer OS cell proliferation and invasion. These functional interactions occur in a dynamic environment of shifting pHi and pHe, which appear to be indirectly modulated via redox signaling through NHE1.
